# Dislocation cluster generation behavior in multicrystalline silicon investigated using twin network analysis

**DOI:** 10.1080/14686996.2025.2512703

**Published:** 2025-05-28

**Authors:** Kazuma Torii, Takuto Kojima, Kentaro Kutsukake, Hiroaki Kudo, Noritaka Usami

**Affiliations:** a Graduate School of Engineering, Nagoya University, Nagoya, Japan; b Department of Energy and Environment Renewable Energy Advanced Research Center, National Institute of Advanced Industrial Science and Technology, Tsukuba, Japan; c Institute of Materials and Systems for Sustainability, Nagoya University, Nagoya, Japan; d Center of Advanced Intelligence Project, RIKEN, Tokyo, Japan; e Graduate School of Informatics, Nagoya University, Nagoya, Japan; f Institutes of Innovation for Future Society, Nagoya University, Nagoya, Japan

**Keywords:** Silicon, dislocation, twin grain boundary, network analysis

## Abstract

We utilized twin network analysis of polycrystalline materials through graph theory to visualize microstructures and examine the behavior of dislocation cluster generation in multicrystalline silicon grown by directional solidification. This approach allows for a rapid and statistical understanding of microstructures and their correlations by representing these features and their changes as network graphs. Our analysis revealed that dislocation clusters are formed at asymmetric Σ27a grain boundaries, which result from a specific twinning process. Gaining this knowledge is expected to assist in identifying grain boundary groups that can minimize the formation of dislocation clusters.

## IMPACT STATEMENT

We utilized graph theory to analyze complex polycrystalline structures, and its application to multicrystalline silicon has visualized the sequential twinning during directional solidification and dislocation cluster generation.

## GRAPHICAL ABSTRACT

**Figure:**
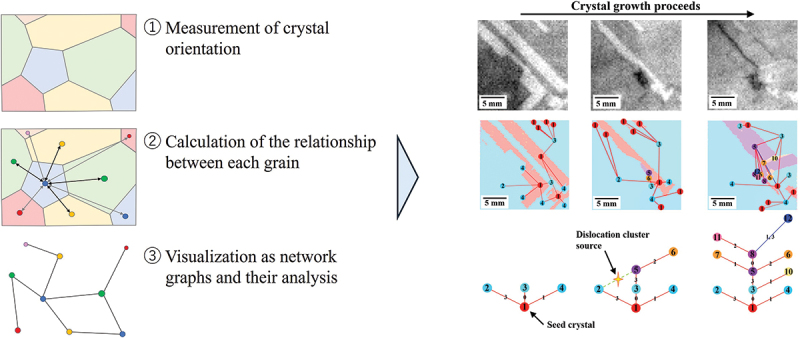

## 1. Introduction

In polycrystalline materials, the presence of crystal defects has a significant effect on the mechanical, electrical, optical, and other properties of the material. For example, increasing the density of dislocations, one type of crystal defect, in cold-worked carbon steel enhances the strength but decreases the ductility [1–6]. Therefore, appropriate design and control of crystal defects for each material are essential for further improvement of the quality and performance of polycrystalline materials [7]. However, polycrystalline materials have a very complicated structure, and various factors such as grain distribution, grain boundaries, orientation, growth process, and crystal defects are intricately related to each other. Therefore, it is very difficult to perform a systematic analysis while simultaneously considering these multiple parameters, and in many polycrystalline materials, the mechanisms of crystal defect generation and logical guidelines for their control have not yet been fully established [8,9].

This issue also applies to multicrystalline silicon, the model material used in this study. It is noted that the term ‘multicrystalline silicon’ is used for the material grown by directional solidification with a grain size of several millimeters or larger instead of ‘polycrystalline silicon’, which is often used for the feedstock for melt growth of the silicon ingot. Silicon is a widely used material for solar cell substrates and other applications due to its abundance and affordability [10,11]. In recent years, monocrystalline silicon has become the dominant material in the solar cell industry, thanks to significant advances in reducing its production costs. However, it is essential to enhance our scientific understanding of multicrystalline silicon as a model material since the knowledge is expected to aid in the development of high-performance polycrystalline materials that could be widely adopted in society.

The electrical properties of solar cells, especially conversion efficiency, tend to be limited by dislocation clusters, in which dislocations as high as 10^7^ cm^−2^ act as recombination centers for photogenerated carriers [10–13]. Therefore, suppression of dislocation clusters is extremely important in multicrystalline silicon. Dislocation cluster generation in multicrystalline silicon has been the focus of extensive research [12–27], and grain boundaries are known to become the source of dislocations. In multicrystalline silicon, there are different types of major grain boundaries. These include random grain boundaries, which are grain boundaries with different growth nuclei, Σ3 grain boundaries, which are twinning boundaries within crystal grains, and Σ3^*n*^ grain boundaries, which are boundaries between crystal grains with different orientations caused by the formation of continuous twins. However, due to the complicated structure unique to multicrystalline silicon, the relationship between microstructures and dislocation cluster generation, especially the role of continuous generation of Σ3^*n*^ grain boundaries, has not yet been fully understood. In order to discuss the relationship between the continuous generation of Σ3^*n*^ grain boundaries and dislocation generation during the growth process, it is useful to visualize the features of complex multicrystalline structures so that they can be intuitively understood. Therefore, we propose to utilize a method called ‘twin network analysis’ to intuitively understand the complex multicrystalline structure sensitively and to investigate the relationship between the crystal structure and the generation of dislocation clusters, using multicrystalline silicon as a model material.

**Figure 1.**
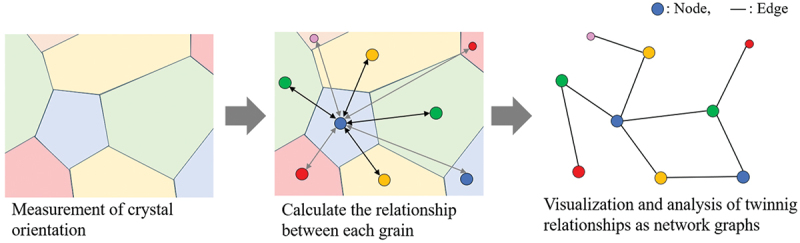
Schematic of twin network analysis.

A schematic diagram of the twin network analysis is shown in Figure 1. The method can extract, visualize, and analyze the relationship of the occurrence of twinning based on graph theory by referring to the crystal orientation information for the rotational quaternions that show the orientation relationship caused by sequential twinning. By conducting twin network analysis, it is possible to investigate the correspondence relationship and growth process of each grain in a multicrystalline structure. In addition, by creating and visualizing network graphs based on the obtained correspondence relationships, it is possible to intuitively and globally grasp complex multicrystalline structures [28,29].

In this paper, the method of twin network analysis is explained in detail, and the results of applying the network analysis to a multicrystalline silicon structure are reported. The relationship between the multicrystalline silicon structure and dislocation clusters are also discussed based on the results of the analysis.

## 2. Experimental methods

### 2.1. Sample preparation and measurement

**Figure 2.**
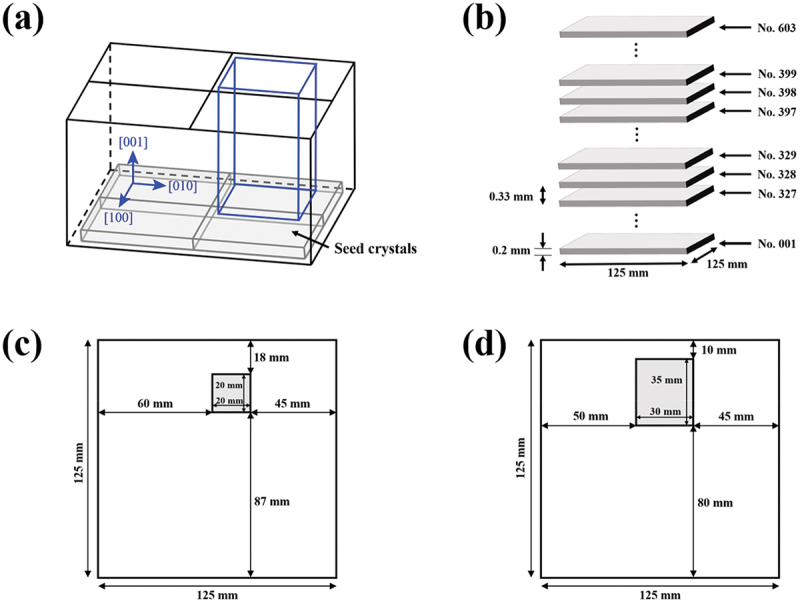
(a) Overall schematic diagram of a multicrystallized silicon ingot sample, (b) schematic diagram of wafers used as samples (blue framed area in Figure. 2(a)), (c) cutout positions of wafers No.327 to No.347, (d) cutout positions of wafers No.351 to No.399.

Silicon ingots that were multicrystallized during the directional solidification process from a single crystal seed with a crystallographic orientation of < 100> in growth directions were used. Details of the sample preparation method are described in Ref [30,31]. A schematic diagram of the entire ingot is shown in Figure 2(a). After cutting out the area indicated by the blue box in Figure 2(a), it was sliced at a pitch of 330 µm and numbered from the bottom as No.1, No.2, up to No.603, as shown in Figure 2(b). In this study, wafers No.327 to No.399, which showed significant microstructural changes due to twinning, were taken out every other 4 wafers and used as samples. In order to satisfy the size limitation for measurements, No.327 to No.347 were cut to 20 mm × 20 mm and No.351 to No.399 to 30 mm × 35 mm with a laser cutter, as shown in Figure 2(c,d).

**Figure 3.**
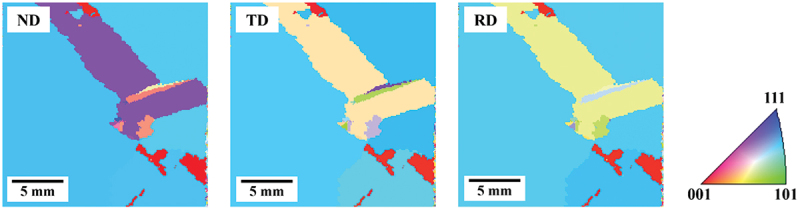
EBSD measurement results and inverse Pole figure.

We collected the physical property data necessary for twin network analysis. Grain distribution and crystal orientation were measured by Electron Back Scatter Diffraction (EBSD) using a Scanning Electron Microscopy (SEM) (JSM-7001FA, JEOL Ltd., Japan). An example of the measurement results is shown in Figure 3. The distribution of dislocation clusters was evaluated using a photoluminescence (PL) imaging system consisting of an InGaAs camera and an excitation light with a wavelength of 940 nm (EPL-100s, Hamamatsu Photonics, Japan). The PL intensity from regions where dislocation clusters are present is lower than that from regions with fewer crystal defects due to nonradiative recombination through defect levels associated with dislocation clusters [32]. In addition, the contrast induced by dislocation clusters is stronger than other defects such as grain boundaries. As a result, regions with dislocation clusters appear relatively darker in the PL image. The areas where the PL intensity is below a certain value are assumed to be dislocation clusters, and the PL image is reconstructed three-dimensionally using ImageJ image processing software to create a cross-sectional view, which enables us to trace the generation of dislocation clusters in three dimensions and to identify the source of dislocation clusters.

### 2.2. Twin network analysis

The twin network analysis can visualize and analyze sequential twin formation in a polycrystalline structure caused by twins by referring to the crystal orientation information for the rotational quaternions. Specifically, the method enables us to investigate the correspondence relationship and growth process of each crystal grain in a polycrystalline structure. In addition, by creating and visualizing network graphs based on the obtained correspondence relationships, it is possible to intuitively and broadly grasp complex polycrystalline structures [28,29].

**Figure 4.**
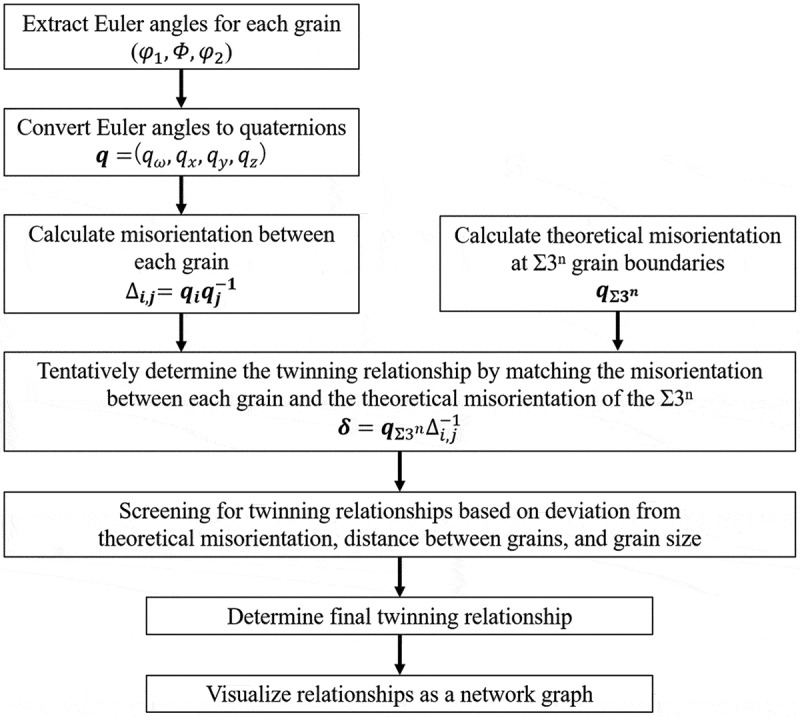
Schematic of twin network analysis.

A schematic diagram of the specific procedure is shown in Figure 4. First, in order to take into account the rotation of grain orientation, the crystal orientation for each grain obtained by EBSD measurement was converted from Euler angle notation ($\varphi_{1},\Phi ,\varphi_{2}$) to quaternion number notation ($q_{\omega },q_{x},q_{y},q_{z}$) by using Eq. (1) [33].

(1) $$\begin{array}{c} q=\left(\begin{array}{c} q_{\omega }\\ q_{x}\\ \begin{array}{c} q_{y}\\ q_{z} \end{array} \end{array}\right)=\left(\begin{array}{c} \cos\frac{\varphi_{1}}{2}\cos\frac{\Phi }{2}\cos\frac{\varphi_{2}}{2}-\sin\frac{\varphi_{1}}{2}\sin\frac{\Phi }{2}\sin\frac{\varphi_{2}}{2}\\ \sin\frac{\varphi_{1}}{2}\cos\frac{\Phi }{2}\cos\frac{\varphi_{2}}{2}+\cos\frac{\varphi_{1}}{2}\sin\frac{\Phi }{2}\sin\frac{\varphi_{2}}{2}\\ \begin{array}{c} \cos\frac{\varphi_{1}}{2}\sin\frac{\Phi }{2}\cos\frac{\varphi_{2}}{2}-\sin\frac{\varphi_{1}}{2}\cos\frac{\Phi }{2}\sin\frac{\varphi_{2}}{2}\\ \sin\frac{\varphi_{1}}{2}\sin\frac{\Phi }{2}\cos\frac{\varphi_{2}}{2}+\cos\frac{\varphi_{1}}{2}\cos\frac{\Phi }{2}\sin\frac{\varphi_{2}}{2} \end{array} \end{array}\right)\# \end{array}$$

Next, using Eqs. (2) and (3), we calculated the misorientation $\Delta_{i,j}$ between arbitrary grains *i* and *j*, and compared it to the theoretical misorientation $q_{\Sigma 3^{n}}$ at the Σ3^*n*^ grain boundary. The one with the smallest difference δ from the misorientation in the theoretical twinning relation was tentatively determined as the twinning relation.

(2) $$\begin{array}{c} \Delta_{i,j}=q_{i}{q_{j}}^{-1}=\left(\begin{array}{ccc} q_{i,\omega } & -q_{i,x} & \begin{array}{cc} -q_{i,y} & -q_{i,z} \end{array}\\ q_{i,x} & q_{i,\omega } & \begin{array}{cc} -q_{i,z} & q_{i,y} \end{array}\\ \begin{array}{c} q_{i,y}\\ q_{i,z} \end{array} & \begin{array}{c} q_{i,z}\\ -q_{i,y} \end{array} & \begin{array}{c} \begin{array}{cc} q_{i,\omega } & -q_{i,x} \end{array}\\ \begin{array}{cc} q_{i,x} & q_{i,\omega } \end{array} \end{array} \end{array}\right)\left(\begin{array}{c} -q_{j,\omega }\\ q_{j,x}\\ \begin{array}{c} q_{j,y}\\ q_{j,z} \end{array} \end{array}\right)\# \end{array}$$

(3) $$\begin{array}{c} \delta =q_{\Sigma }{\Delta_{i,j}}^{-1}=\left(\begin{array}{ccc} q_{\Sigma ,\omega } & -q_{\Sigma ,x} & \begin{array}{cc} -q_{\Sigma ,y} & -q_{\Sigma ,z} \end{array}\\ q_{\Sigma ,x} & q_{\Sigma ,\omega } & \begin{array}{cc} -q_{\Sigma ,z} & q_{\Sigma ,y} \end{array}\\ \begin{array}{c} q_{\Sigma ,y}\\ q_{\Sigma ,z} \end{array} & \begin{array}{c} q_{\Sigma ,z}\\ -q_{\Sigma ,y} \end{array} & \begin{array}{c} \begin{array}{cc} q_{\Sigma ,\omega } & -q_{\Sigma ,x} \end{array}\\ \begin{array}{cc} q_{\Sigma ,x} & q_{\Sigma ,\omega } \end{array} \end{array} \end{array}\right)\left(\begin{array}{c} -\Delta_{i,j,\omega }\\ \Delta_{i,j,x}\\ \begin{array}{c} \Delta_{i,j,y}\\ \Delta_{i,j,z} \end{array} \end{array}\right)\# \end{array}$$

The final twinning relationship was then determined by deleting and recalculating the twinning relationships for those with large deviations δ from the theoretical misorientation $q_{\Sigma 3^{n}}$, large distances between grains, and extremely small grain sizes. Finally, a network graph was created by connecting grains in a twinning relationship centered on the Σ3 correspondence among the twinning relationships that were determined. The following attributes were added to the nodes and edges when creating the network graph to incorporate more detailed information on the twin formation process and dislocation clustering points:

**(1) Node color: crystal orientation**

Node colors were assigned for each grain orientation, corresponding to the vertical direction in the inverse pole figure. Therefore, the crystal orientation can be intuitively grasped by checking the colors of the nodes on the network graph.

**(2)Node number: Grain ID**

Nodes were assigned a Grain ID obtained by EBSD measurement. Therefore, the position of a grain can be confirmed by checking the number of nodes on the network graph. For simplification, grains with the same crystal orientation are collapsed to the one with the youngest Grain ID on the network graph.

**(3)Edge color: Twinning relationship**

The color of the edges in the graph represents the twinning relationship between each grain. This means that by looking at the edge color, we can confirm the twinning relationship between grains. The graph connects the nodes with red edges to indicate the Σ3 grain boundary, blue edges for the Σ9 grain boundary, and green edges for the Σ27a grain boundary. The colors other than red are used when the intervening grains disappear in the growth process.

**(4)Edge color: Twinning rotation axis**

**Figure 5.**
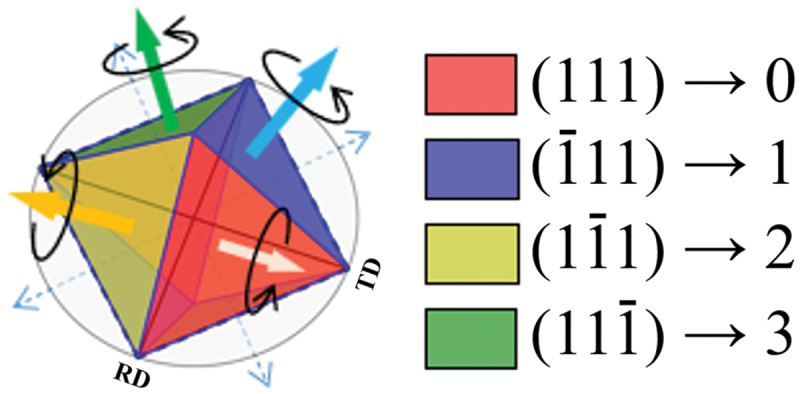
Four twin rotation axes and corresponding edge numbers.

The Σ3 grain boundary with the four {111} planes as the axis of rotation represents the fundamental twinning relationship of multicrystalline silicon. This relationship corresponds to the numbers 0 to 3 as shown in Figure 5, with each edge being assigned the number from 0 to 3. By checking the edge numbers, detailed twinning relationships between grains can be confirmed. The twin grain boundary represented by Σ3^*n*^ can also be expressed in the same way by a combination of the numbers 0 to 3, since the twin boundary is caused by continuous twinning of Σ3 grain boundary. (e.g. Σ9 grain boundary: [0,1] [2,3], etc.; Σ27a grain boundary: [0,1,0] [1,3], etc.)

**(5)Dashed lines and stars: Dislocation cluster occurrence points**

Based on the position of dislocation cluster occurrence obtained through PL imaging, dashed lines were connected between the nodes indicating grains with grain boundaries corresponding to the dislocation cluster origin, and stars were marked. The color of the dashed lines corresponds to the twinning relationship as well as that of the edges. Therefore, by checking the dashed lines and stars, the location of dislocation cluster occurrence and grain boundary information can be confirmed.

Based on the above, we analyzed the behavior of dislocation cluster generation by observing the process of the development of the created twin network as it changes along with crystal growth, comparing it with other measurement data. It is noted that while the above features were used in this study, other features including grain size, misorientation, stress distribution, etc. can be incorporated into the graph if the data are available.

## 3. Results and discussions

### 3.1. Results

**Figure 6.**
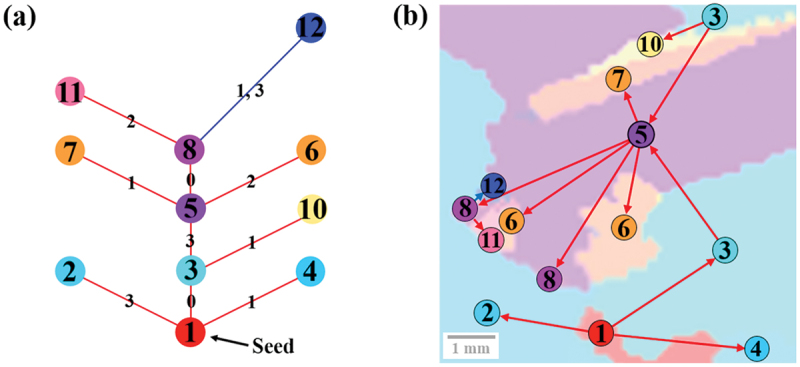
(a) Twin network graph and (b) IPF color map with twin network graph superimposed.

Figure 6 shows an example of a grain boundary network analysis. In general, higher-order corresponding grain boundaries are caused by a sequence of twins, so by looking at the twin network graph, we can intuitively grasp the growth process of which grain originated from which grain. In Figure 6, it can be seen that twins are continuously generated from the seed crystal, grain 1 (red). Three grains 2, 3, and 4, each with a Σ3 correspondence based on a different rotation axis, are twinned to grain 1, which is the seed crystal, and grain 5, a twinned grain with the $(11\overset{ˉ}{1}$) plane as its rotation axis, is generated from grain 3. Thus, by looking at the graph, the grain growth process can be intuitively traced. By looking at the graph, one can understand that the sequential twinning rather than the collision of the crystal grains is responsible for the multicrystallization in this sample. In addition, it is seen that the sequential twinning has been observed up to the fifth order. Thus, by using the grain boundary network graph, we can intuitively understand the growth process of the microstructure and crystallographic microstructural relationships in polycrystalline materials.

**Figure 7.**
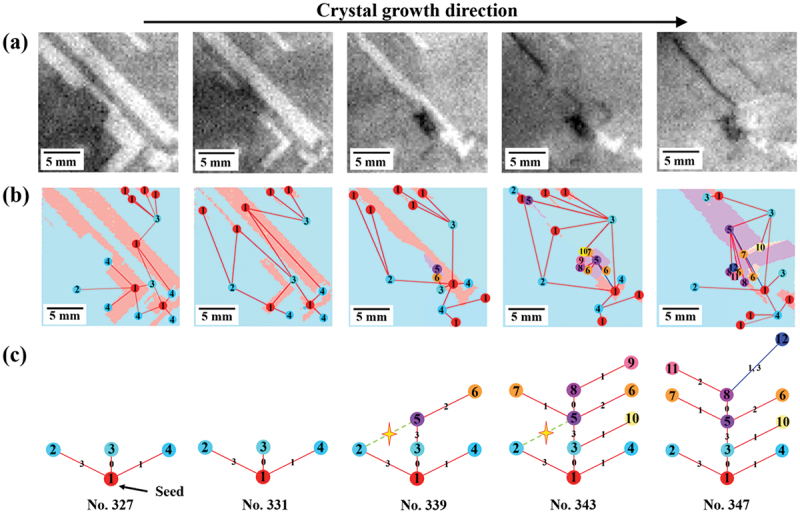
(a) PL image, (b) inverse Pole figure map + superimposed twin network graph, and (c) twin network graph at the point of dislocation cluster generation.

**Figure 8.**
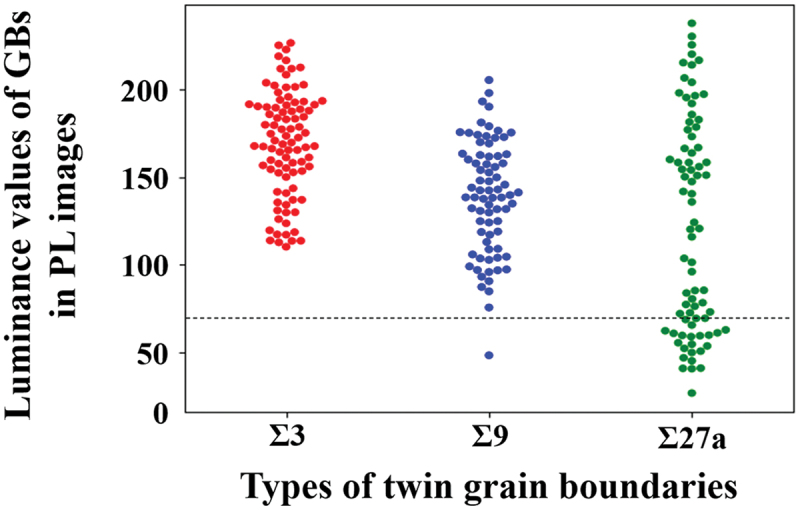
Luminance value distribution of PL images at mainly observed twin grain boundaries. Dislocation clusters were defined as those with luminance values below 70.

The method was used to identify crystallographic microstructural features related to dislocation cluster generation. Figure 7 shows an example of data associated with crystal growth near the location where dislocation clusters generate: (a) PL image, (b) inverse pole Figure map superimposed with twin network graph, and (c) twin network graph. From the entire Figure 7, the structural changes associated with crystal growth and the corresponding development of twin network graphs and dislocation clusters can be observed. In particular, the twin network graph shows that the twins rapidly develop into the fifth order twins in a short period of time, during which dislocation clusters are also generated. These figures are then examined in more detail. Figure 7(a) shows the generation of dislocation clusters associated with crystal growth. The particularly black-colored areas in the three images on the right side are the locations of dislocation clusters. By comparing this area with Figure 7(b), we can see the generation of dislocation clusters near the grain boundaries of grain 2 (light blue) and grain 5 (purple). When we examine these grains on the twin network graph depicted in Figure 7(c), we observe that they align with the Σ27a grain boundary, which is separated by three edges ([3,0,3]) containing the same rotation axis. This pattern was also observed in other dislocation clustering locations, with the majority of dislocation clusters stemming from the Σ27a grain boundary. Figure 8 shows the main grain boundaries identified in this study and the distribution of luminance values for each grain boundary in the PL image. The areas with the luminance values of 70 or less were defined as dislocation clusters. It is evident that the grain boundaries with luminance values below 70 are primarily concentrated at the Σ27a grain boundary, indicating the presence of dislocation clusters. These phenomena are consistent with the results of previous studies, and the factors behind them are discussed in the next section [23,34,35].

## 4. Discussions

**Figure 9.**
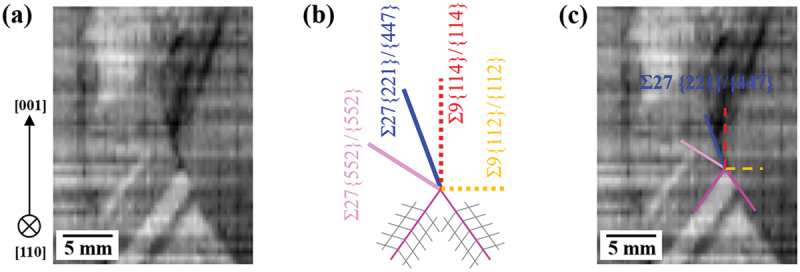
Dislocation cluster generation from asymmetric Σ27a grain boundaries. (a) Cross-section of 3D reconstructed PL image around the point of dislocation cluster generation, (b) theoretical growth direction of each twin grain boundary [33], (c) superposition of (a) and (b) along the Σ27a grain boundary where the dislocation cluster exists.

Dislocation clustering from Σ27a grain boundaries is discussed in terms of grain boundary energies. Table I shows the grain boundary energies of twin grain boundaries in silicon (the calculation method is described in Ref [25]). Asymmetric Σ27a grain boundaries are more likely to be generated because the difference in grain boundary energy between symmetric and asymmetric Σ27a grain boundaries is very small. Figure 9(a) shows a 2D cross-section of the 3D reconstructed PL image near the Σ27a grain boundary, where the generation of dislocation clusters represented by the black areas was observed. It is seen that dislocation clusters are generated along the Σ27a grain boundary. Figure 9(b) shows a schematic diagram of the direction in which each twin grain boundary occurs with respect to the Σ3 grain boundary. Figure 9(c) is an overlay of Figure 9(a,b). From Figure 9(c), the Σ27a grain boundary where dislocation clusters are generated coincides with the asymmetric grain boundary Σ27a{221}/{447}, not the symmetric grain boundary Σ27a{552}/{552}. It should be noted that asymmetric Σ27a grain boundaries were observed at many of the locations where dislocation clusters were present. In general, asymmetric grain boundaries are prone to local stress concentration from the viewpoint of non-uniformity of lattice arrangement, and this is considered to be the origin of dislocation clusters [36].

**Table 1.** Grain boundary energy at symmetric/asymmetric Σ27a grain boundaries [32].

Type of grain boundary	Grain boundary energy
Σ27a {552}/{552} (symmetric)	0.336 J/m^2^
Σ27a {221}/{447} (asymmetric)	0.346 J/m^2^

**Figure 10.**
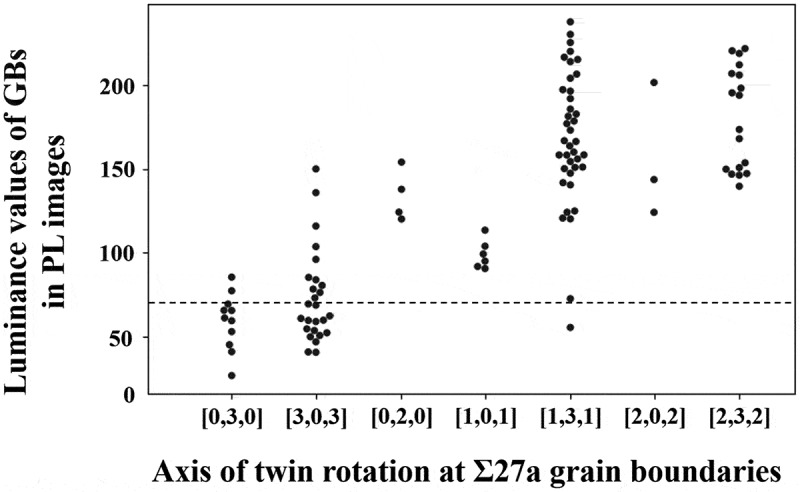
The presence or absence of dislocation clusters based on differences in the twin rotation axis. (see Figure. 4 for the twin rotation axis.).

The finding from Figure 8 indicates that even within the Σ27a grain boundaries, there are variations in the generation of dislocation clusters. To investigate the reason, based on the results of the network graph analysis, each Σ27a grain boundary was further subdivided in terms of the difference in its twinning process. It is noted that although the Σ27a grain boundaries can be classified into 16 patterns based on differences in the twinning rotation axis, we were unable to identify all patterns due to sample and analysis coverage issues. The results, shown in Figure 10, revealed that dislocation clusters were observed only from grain boundaries with specific twinning processes, even within the Σ27a grain boundaries. The different twinning processes led to variations in grain boundary direction and orientations. Multicrystalline silicon is subjected to a certain amount of stress from the crucible wall due to solidification and expansion during manufacturing, but the magnitude of the stress varies depending on the direction and orientation of grain boundaries [37]. In fact, previous research has suggested that depending on the direction and orientation of grain boundaries, stress is locally concentrated on certain grain boundaries, resulting in the generation of dislocation clusters [25]. Since there is a close relationship between the direction and orientation of grain boundaries and stress, these differences in the twinning process may have resulted in the presence or absence of local stress concentration and contributed to the generation of dislocation clusters (Figure 10).

**Figure 11.**
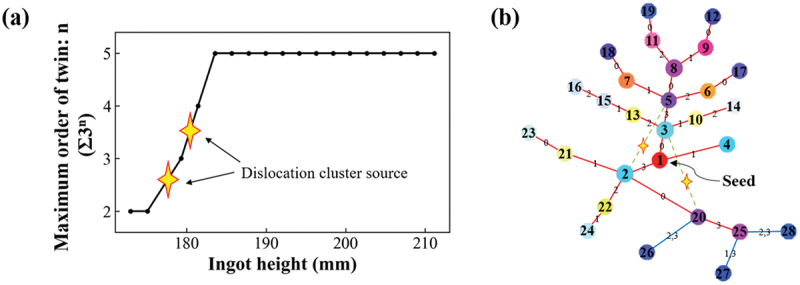
(a) Maximum order of twinning of the grains in the wafer relative to the seed crystal at each ingot height, (b) final twin network graph and summary of dislocation cluster generation grain boundaries. (Grain boundaries where some dislocation clusters occur overlap).

**Figure 12.**
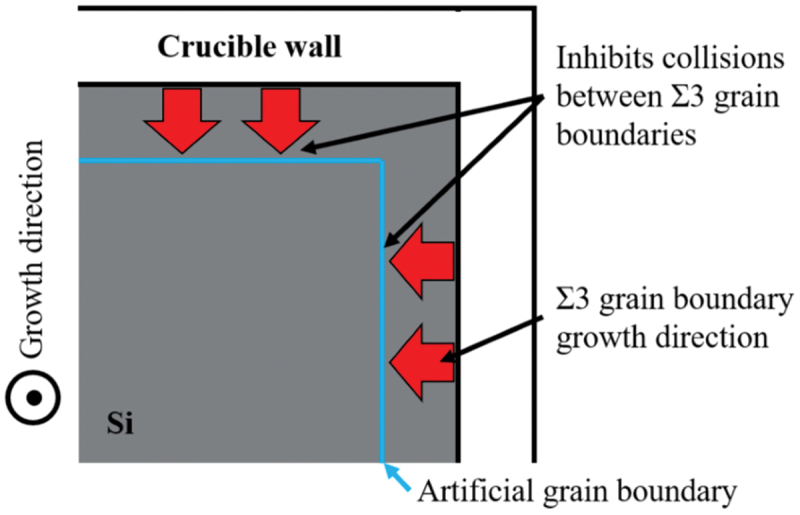
Suppression of dislocation clusters using artificial grain boundaries.

Based on these results, we will discuss how to control dislocation clusters. Figure 11(a) shows the process of twin development and the locations where dislocation clusters are generated, and Figure 11(b) shows the final twin network graph and the generation of dislocation clusters. Figure 11(a) shows that the twins rapidly develop into the fifth order twins in a short period of time (less than 1 cm) and then stabilize, indicating the generation of dislocation clusters during this rapid twin development. The network graph in Figure 11(b) shows that the generation of dislocation clusters occurs mainly near the seed crystal, i.e. between low-order twin grains. Therefore, suppression of dislocation clusters can be expected by suppressing the formation of Σ27a arising from between low-order twin grains in the initial stage of twin formation. In fact, a method to suppress multicrystallization using artificial grain boundaries has been proposed [38]. We believe that dislocation clusters can be suppressed by applying this method and providing seed crystals with arbitrary artificial grain boundaries. By providing the seed crystal with arbitrary artificial grain boundaries as shown in Figure 12, we can suppress the collision of Σ3 grain boundaries in the initial stage and the subsequent generation of Σ27a grain boundaries.

## 5. Conclusions

We applied twin network graphs to visualize and analyse the complex multicrystalline structures to intuitively understand the growth process and crystallographic structural relationships. In addition, the evaluation of crystallographic structural features related to dislocation cluster generation in multicrystalline silicon revealed that dislocation clusters tend to be generated from asymmetric Σ27a grain boundaries that arise from low-order twin grains. Although we focused on the twinning relationship in this study, the network analysis will be useful for visualization and analysis of other features and microstructural relationships, as well as for application to graph neural networks, which have been attracting increasing attention in recent years. We believe that this method will contribute to the analysis of polycrystalline materials as well as to the further improvement of the quality and performance of polycrystalline materials in the future.

## References

[1] Meyers MA, Mishra A, Benson DJ. Mechanical properties of nanocrystalline materials. Prog Mater Sci. 2006;51(4):427–10. doi: 10.1016/j.pmatsci.2005.08.003

[2] Li J, Porter L, Yip S. Atomistic modeling of finite-temperature properties of crystalline β-SiC: II. Thermal conductivity and effects of point defects. J Nucl Mater. 1998;255(2–3):139–152. doi: 10.1016/S0022-3115(98)00034-8

[3] Alkauskas A, McCluskey MD, Van de Walle CG. Tutorial: defects in semiconductors—combining experiment and theory. J Appl Phys. 2016;119(18):181101. doi: 10.1063/1.4948245

[4] Gil Sevillano J, van Houtte P, Aernoudt E. Large strain work hardening and textures. Prog Mater Sci. 1980;25(2–4):69–134. doi: 10.1016/0079-6425(80)90001-8

[5] Kumar KS, Van Swygenhoven H, Suresh S. Mechanical behavior of nanocrystalline metals and alloys. Acta Mater. 2003;51(19):5743–5774. doi: 10.1016/j.actamat.2003.08.032

[6] Qu Z, Sparks TD, Pan W, et al. Thermal conductivity of the gadolinium calcium silicate apatites: effect of different point defect types. Acta Mater. 2011;59(10):3841–3850. doi: 10.1016/j.actamat.2011.03.008

[7] Li M, Zhang CT, Li MY, et al. Growth defects of organic crystals: a review. Chem Eng J. 2022;429:132450. doi: 10.1016/j.cej.2021.132450

[8] Hara K, Kojima T, Kutsukake K, et al. A machine learning-based prediction of crystal orientations for multicrystalline materials. APL Mach Learn. 2023;1(2):026113. doi: 10.1063/5.0138099

[9] Hara K, Kojima T, Kutsukake K, et al. 3D CNN and grad-CAM based visualization for predicting generation of dislocation clusters in multicrystalline silicon. APL Mach Learn. 2023;1(3):036106. doi: 10.1063/5.0156044

[10] Möller HJ, Funke C, Rinio M, et al. Multicrystalline silicon for solar cells. Thin Solid Films. 2005;487(1–2):179–187. doi: 10.1016/j.tsf.2005.01.061

[11] Castellanos S, Ekstrom KE, Autruffe A, et al. High-performance and traditional multicrystalline silicon: comparing gettering responses and lifetime-limiting defects. IEEE J Photovolt. 2016;6(3):632–640. doi: 10.1109/JPHOTOV.2016.2540246

[12] Sopori B, Rupnowski P, Mehta V, et al. Performance limitations of mc-Si solar cells caused by defect clusters. ECS Trans. 2009;18(1):1049–1058. doi: 10.1149/1.3096571

[13] Kveder V, Kittler M, Schroter W. Recombination activity of contaminated dislocations in silicon: a model describing electron-beam-induced current contrast behavior. Phys Rev B. 2001;63(11):115208. doi: 10.1103/PhysRevB.63.115208

[14] Ryningen B, Stokkan G, Kivambe M, et al. Growth of dislocation clusters during directional solidification of multicrystalline silicon ingots. Acta Mater. 2011;59(20):7703–7710. doi: 10.1016/j.actamat.2011.09.002

[15] Oriwol D, Trempa M, Sylla L, et al. Investigation of dislocation cluster evolution during directional solidification of multicrystalline silicon. J Cryst Growth. 2017;463:1–9. doi: 10.1016/j.jcrysgro.2017.01.027

[16] Takahashi I, Usami N, Kutsukake K, et al. Generation mechanism of dislocations during directional solidification of multicrystalline silicon using artificially designed seed. J Cryst Growth. 2010;312(7):897–901. doi: 10.1016/j.jcrysgro.2010.01.011

[17] Zhou N, Sui X, He X, et al. Nucleation of self-growth dislocations on growth front during the solidification process of silicon. J Appl Phys. 2019;125(15):155108. doi: 10.1063/1.5088125

[18] Trempa M, Kupka I, Kranert C, et al. Evolution of grain structure and recombination active dislocations in extraordinary tall conventional and high performance multi-crystalline silicon ingots. J Cryst Growth. 2017;459:67–75. doi: 10.1016/j.jcrysgro.2016.11.030

[19] Stokkan G, Hu Y, Mjøs Ø, et al. Study of evolution of dislocation clusters in high performance multicrystalline silicon. Sol Energy Mater Sol Cells. 2014;130:679–685. doi: 10.1016/j.solmat.2014.02.034

[20] Autruffe A, Hagen VS, Arnberg L, et al. Dislocation generation at near-coincidence site lattice grain boundaries during silicon directional solidification. J Cryst Growth. 2015;411:12–18. doi: 10.1016/j.jcrysgro.2014.10.054

[21] Nakano S, Chen XJ, Gao B, et al. Numerical analysis of cooling rate dependence on dislocation density in multicrystalline silicon for solar cells. J Cryst Growth. 2011;318(1):280–282. doi: 10.1016/j.jcrysgro.2010.11.009

[22] Takahashi I, Joonwichien S, Matsushima S, et al. Relationship between dislocation density and contact angle of dendrite crystals in practical size silicon ingot. J Appl Phys. 2015;117(9):095701. doi: 10.1063/1.4913855

[23] Stokkan G, Song A, Ryningen B. Investigation of the grain boundary character and dislocation density of different types of high performance multicrystalline silicon. Crystals. 2018;8(9):341. doi: 10.3390/cryst8090341

[24] Ohno Y, Tajima K, Kutsukake K, et al. Generation of dislocation clusters at triple junctions of random angle grain boundaries during cast growth of silicon ingots. Appl Phys Express. 2020;13(10):105505. doi: 10.35848/1882-0786/abbb1c

[25] Yamakoshi K, Ohno Y, Kutsukake K, et al. Multicrystalline informatics applied to multicrystalline silicon for unraveling the microscopic root cause of dislocation generation. Adv Mater. 2024;36(8):2308599. doi: 10.1002/adma.202308599 38041569

[26] Oriwol D, Hollatz M, Reinecke M. Control of dislocation cluster formation and development in silicon block casting. Energy Procedia. 2012;27:66–69. doi: 10.1016/j.egypro.2012.07.030

[27] Oriwol D, Carl ER, Danilewsky AN, et al. Small-angle subgrain boundaries emanating from dislocation pile-ups in multicrystalline silicon studied with synchrotron white-beam X-ray topography. Acta Mater. 2013;61(18):6903–6910. doi: 10.1016/j.actamat.2013.08.002

[28] Reed BW, Kumar M. Mathematical methods for analyzing highly-twinned grain boundary networks. Scr Mater. 2006;54(6):1029–1033. doi: 10.1016/j.scriptamat.2005.11.045

[29] Usami N, Kutsukake K, Kojima T, et al. Multicrystalline informatics: a methodology to advance materials science by unraveling complex phenomena. Sci Technol Adv Mater. 2024;25(1):2396272. doi: 10.1080/14686996.2024.2396272 39308887 PMC11413958

[30] Riepe S, Krenckel P, Hayama Y, et al. Enhanced material quality in smart mono-Si block cast ingots by introduction of functional defects. In: 36^th^ European Photovoltaic Solar Energy Conference and Exhibition; Marseille, France; 2019. doi: 10.4229/EUPVSEC20192019-2AO.5.3

[31] Krenckel P, Hayama Y, Schindler F, et al. Propagation of crystal defects during directional solidification of silicon via induction of functional defects. Crystals. 2021;11(2):90. doi: 10.3390/cryst11020090

[32] Trupke T, Mitchell B, Weber JW, et al. Photoluminescence imaging for photovoltaic applications. Energy Procedia. 2012;15:135–146. doi: 10.1016/j.egypro.2012.02.016

[33] Huynh DQ. Metrics for 3D rotations: comparison and analysis. J Math Imag Vis. 2009;35(2):155–169. doi: 10.1007/s10851-009-0161-2

[34] Oliveira VA, Marie B, Cayron C, et al. Formation mechanism and properties of twinned structures in (111) seeded directionally solidified solar grade silicon. Acta Mater. 2016;121:24–36. doi: 10.1016/j.actamat.2016.08.063

[35] Tsoutsouva MG, Riberi-Béridot T, Regula G, et al. In situ investigation of the structural defect generation and evolution during the directional solidification of 〈110〉 seeded growth Si. Acta Mater. 2016;115:210–223. doi: 10.1016/j.actamat.2016.06.004

[36] Kaneko H. Structure of grain boundary. Tetsu-to-Hagané [Iron And Steel]. 1970;56(5):118–131. doi: 10.2355/tetsutohagane1955.56.5_622

[37] Kamaya M, Kawamura Y, Kitamura T. Three-dimensional local stress analysis on grain boundaries in polycrystalline material. Int J Solids Struct. 2007;44(10):3267–3277. doi: 10.1016/j.ijsolstr.2006.09.020

[38] Kutsukake K, Usami N, Ohno Y, et al. Control of grain boundary propagation in mono-like Si: utilization of functional grain boundaries. Appl Phys Express. 2013;6(2):025505. doi: 10.7567/APEX.6.025505

